# Case of the Removal of a Diseased Ovarium, Terminating Fatally on the Seventh Day after the Operation

**Published:** 1844-04-06

**Authors:** J. M. Greenhow

**Affiliations:** Surgeon to the Newcastle Infirmary, and Eye Infirmary


### RECORD OF MEDICAL SCIENCE.

*Case of the Removal of a Diseased Ovarium, terminating fatally on the seventh day after the operation.* By J. M. Greenhow, Esq., Surgeon to the Newcastle Infirmary, and Eye Infirmary. [Communicated by Sir B. C. Brodie, Bart.

The patient was aged 29 years, and married. For four years she had suffered from frequent discharges of blood from the uterus. Eighteen months ago—that is, six months after her marriage—the swelling in the abdomen commenced at the pubic region, and rapidly increased till it attained a large size; her strength all the while declining from the constant uterine discharge. The abdomen was tapped, and only a little blood escaped; but afterwards nearly a quart of dark-coloured fluid was discharged daily from the wound for about a fortnight. Before the operation of removing the tumour, which was performed September 3d, the abdomen was about as large as at the full period of utero-gestation; there was fluctuation in one or two parts, but generally the tumour was firm, and felt as if divided into separate masses. The incision reached from a little below the ensiform cartilage to near the pubis. Several adhesions existed at different parts, the principal one being to the omentum, which was spread over the upper part of the right side of the tumour. These adhesions were divided with the bistoury, and then the tumour was raised, with some effort, owing to its great size and weight, from its situation. Double ligatures were passed through its pedicle, and firmly tied; and this part being divided near the tumour, it was liberated from its attachments and removed. Two arteries bled freely, one in the divided omentum, and the other in the pedicle; upon these being secured, the wound was brought together by sutures and adhesive plaster, and a bandage applied.

The operation was well borne by the patient, although she vomited several times towards the end. The quantity of blood lost did not exceed six ounces. The symptoms which followed were chiefly great retching and vomiting, constipation of the bowels, quick pulse, tenderness and distension of the abdomen; and she died on the morning of the seventh day. On the post-mortem examination, the folds of the intestines and the omentum were found glued together by recently effused lymph; there was inflammatory redness, with points of ulceration near the pyloric orifice of the stomach. The uterus was healthy, but its cavity was lined with a vascular membrane like the decidua. The morbid growth had been attached to the left broad ligament. On examining the tumour it was found to weigh twelve pounds seven ounces, and to be more than two feet in circumference. Its surface was smooth, and of a pale colour, resembling that of the skin. With the exception of a few cysts containing a yellow fluid, the general mass was composed of a dense vascular cellular structure, with a number of small cells or cysts pervading its substance. The author concluded by making some remarks on the case; he directed attention particularly to the disease found in the stomach, which he considered had an important influence in leading to the fatal termination; and thought, on the whole, that the result of the case did not offer a strong argument against resorting to a similar operation in such cases.

